# The role of body and environment in cognition

**DOI:** 10.3389/fpsyg.2013.00465

**Published:** 2013-07-22

**Authors:** Dermot Lynott, Louise Connell, Judith Holler

**Affiliations:** ^1^Embodied Cognition Lab, Decision and Cognitive Sciences Research Centre, Manchester Business School, University of ManchesterManchester, UK; ^2^Embodied Cognition Lab, School of Psychological Sciences, University of ManchesterManchester, UK; ^3^Language and Cognition Department, Max Planck Institute for Psycholinguistics, NijmegenNijmegen, Netherlands; ^4^School of Psychological Sciences, University of ManchesterManchester, UK

In this Research Topic, we aimed to develop our understanding of cognition by considering the diverse and dynamic relationship between the language we use, our bodily perceptions, and our actions and interactions in the broader environment. We received twenty-six articles that take very different approaches to exploring the question of how our bodies and the environment influence cognition.

Several papers examine how perceptual concepts are developed and accessed. Gainotti (2012) reviews evidence from cognitive neuropsychology and proposes that different types of concepts differentially rely on sensorimotor experience, with somatosensory and movement information playing a major role in artifact representations and visual and other perceptual information playing a major role in the representation of living things. Krause et al. (2013) find an interference effect between fingers and numbers in a numerosity comparison task and suggest that it emerges from an embodied representation of number based on a shared metric for symbolic and tactile numerosities. Since perceptual stimulation sometimes interferes with and sometimes facilitates other conceptual processing Connell and Lynott (2012), review recent findings and propose that these differences arise due to the attentional demands on modality-specific processing. Two groups use event-related potentials to examine how perceptual information is accessed in conceptual tasks. Hald et al. (2013) find evidence for modality-specific grounded representations when processing negated sentences, and demonstrate differential modulation of the N400 according to whether or not a true vs. false sentence involves modality switching. Louwerse and Hutchinson (2012) show that different tasks rely on linguistic vs. perceptual information to different extents, with activation in linguistic cortical regions preceding activation in perceptual cortical regions when both types of processing were associated with the task.

As well as perceptual information, motor information relating to action concepts was also a central topic. In a review of behavioral and neuroimaging work on semantics across different domains (e.g., concrete/abstract words, numbers, and arithmetic), Hauk and Tschentscher (2013) argue that the specific function of sensorimotor areas in processing meaning remains unclear, and suggest that only by employing a combination of methods can causal underpinnings be deduced. However, in their review, Tomasino and Rumiati (2013) contend that the strategy a participant employs in a task is more important than the nature of the stimulus in determining whether motor simulations will be activated and support the view that the motor system is implicated in—but not necessary to—semantic processing. Locatelli et al. (2012) provide evidence for the role of motor experience in motor semantics by demonstrating that action experience in the form of manual dexterity training facilitated subsequent performance in judging sentence-picture pairs that were related to the previously-learned actions. Motor semantics also depend on the time at which an action is described as taking place. Anderson et al. (2013) found that changing the grammatical aspect of action verbs (e.g., *walking* vs. *walked*) caused people to represent events at different levels of detail according to whether event descriptions were set in the recent or distant past.

Perception and action, of course, interact. In a novel use of a Wii balance board, Haazebroek et al. (2013) asked people to imagine they were on either a snowboard or skis and found that this imagined difference mediated a Simon effect, which they subsequently simulated in the HiTEC connectionist model, and suggest a tight coupling exists between perception/action and higher-level cognition. Action execution is also affected by what one knows about a target object: Asai et al. (2012) showed that the knowledge of whether a ball weighed 1kg (vs. 130 g) caused participants to raise their arms above the horizontal in response to an image of a hand holding the ball. They propose that this “heaviness contagion” emerges automatically due to mandatory simulation of others' sensations. Fukui and Inui (2013) demonstrated that whether or not participants could see their own hand when pantomiming a grasp action affected variability and aperture of the executed grasp, and argue that the dorsal stream, as well as the ventral stream, is involved in pantomimed action.

The body and environment interact extensively in spatial cognition. Crollen and Collignon (2012) review how visually-deprived individuals develop representations of spatial frames of reference and propose that sighted people learn to recode spatial information to an external reference frame (i.e., independent of limb/body position) as opposed to the internal reference frame (i.e., dependent on limb/body position) preferred by those without vision. Johannsen and de Ruiter (2013) observed that people's reference frame selection during scene processing is affected by the realism of the scene, with people more likely to choose an egocentric frame of reference when the background is more realistic. They suggest that greater realism results in easier perceptual simulation and therefore a greater preference for egocentric processing. Two separate articles focused on examining how abstract spatial terms may be grounded in concrete spatial experience. Tower-Richardi et al. (2012) demonstrated a correspondence between abstract absolute frames of reference (e.g., *north, east*) and relative body-centered frames of reference (*up, left*): people performed longer hand movements toward relative targets when primed with incongruent absolute terms (e.g., *north* priming *left*). Dijkstra et al. (2012), on the other hand, showed that even metaphorical space is affected by bodily perceptions. In a study using Wii balance boards, they found that when participants unconsciously leaned to the left or right, they attributed more political statements to congruent left-leaning or right-leaning political parties.

Several articles point to the interplay between body and emotion. Havas and Matheson (2013) provide a theoretical perspective on the importance of bodily feedback in the representation of emotions and understanding of emotional language, and argue that bodily states can facilitate the simulation of emotional content during language processing. Kret et al. (2013) demonstrate that emotion recognition depends not only on others' faces, but also on others' bodies. Participants were sensitive to the congruency of emotions expressed by paired bodies and faces, but emotional responses to these stimuli were also mediated by individual differences in anxiety. Furthermore, where previous work has demonstrated that emotional valence judgments (e.g., *right is good, left is bad*) are body-specific, Kominsky and Casasanto (2013) showed that such evaluations can also depend on the abilities of other people's bodies when we reason from their perspective.

As well as taking other people's bodies into account, people are also highly sensitive to where other people are looking. Knoeferle and Kreysa (2012) found that listeners rapidly respond to shifts in speaker's gaze in affecting not only their allocation of visual attention, but also their processing of syntactic structures and assignment of thematic roles, even when such information is not central to the task. Additionally, Pfeiffer and colleagues (Pfeiffer et al., 2012) used a novel interactive eye-tracking paradigm to show that both congruency and latency of an interaction partner's gaze behavior influence one's experience of agency, and that shared attention takes longer to establish than joint attention.

While the majority of articles focus on typical embodiment, two contributions focus on examples of atypical embodiment. Eigsti (2013) provides a review of embodiment in autism spectrum disorders (ASD), and suggests that deficits in co-ordinating motor and conceptual information may result in under-embodiment in individuals with ASD. Lewis et al. (2013) investigated phantom limb experience in non-amputees using a variation on the rubber hand illusion. They found that participants experienced a sense of presence of a “missing” finger, and even described specific sensations (e.g., tingling), suggesting that phantom limb experiences may be an example of over-embodiment where peripersonal perception is folded into body representations.

Finally, a number of contributions consider future directions for the field of embodied cognition. Madan and Singhal (2012) ask the question that, if the body affects cognition, could exercising the body enhance cognition? They draw on diverse literature including work on gesture, memory, and physical exercise, and suggest that a much more integrative approach is needed to determine how movement and exercise may boost cognitive performance. Willems and Francken (2012) contend that, while there is good general support for theories of embodied cognition, too often underspecified theories can generate opposing predictions for the same phenomenon. As such, embodied theories should be capable of providing more specific hypotheses to elucidate exactly when and how the body and environment affect cognition. Wilson and Golonka (2013), however, suggest that body and environment are constantly affecting cognition. They consider whether mental representations are at all necessary to cognitive function in their support of the replacement hypothesis of cognition, which puts the focus firmly on the interaction between an organism and the rich and varied information provided by the environment.

In highlighting the diversity of perspectives and approaches current in embodied cognition research, these articles paint a picture of a field that has matured significantly in recent years. We hope this Research Topic opens up new avenues and challenges for future work on the interplay between cognition, body, and environment.
